# Electromagnetic Feet With Soft Toes for Adaptive, Versatile, and Stable Locomotion of an Inchworm-Inspired Pipe Crawling Robot

**DOI:** 10.3389/fbioe.2022.842816

**Published:** 2022-02-18

**Authors:** Muhammad Bilal Khan, Thirawat Chuthong, Jettanan Homchanthanakul, Poramate Manoonpong

**Affiliations:** ^1^ Bio-inspired Robotics and Neural Engineering Lab, School of Information Science and Technology, Vidyasirimedhi Institute of Science and Technology, Rayong, Thailand; ^2^ Embodied AI and Neurorobotics Lab, SDU Biorobotics, The Mæsk Mc-Kinney Møller Institute, University of Southern Denmark, Odense M, Denmark

**Keywords:** hybrid foot, soft robot, silicone elastomers, bio-inspired legged robot, locomotion control, magnetic adhesion, inspection robot

## Abstract

Feet play an important role in the adaptive, versatile, and stable locomotion of legged creatures. Accordingly, several robotic research studies have used biological feet as the inspiration for the design of robot feet in traversing complex terrains. However, so far, no robot feet can allow legged robots to adaptively, versatilely, and robustly crawl on various curved metal pipes, including flat surfaces for pipe inspection. To address this issue, we propose here a novel hybrid rigid-soft robot-foot design inspired by the leg morphology of an inchworm. The foot consists of a rigid section with an electromagnet and a soft toe covering for enhanced adhesion to a metal pipe. Finite element analysis , performed under different loading conditions, reveals that due to its compliance, the soft toe can undergo recoverable deformation with adaptability to various curved metal pipes and plain metal surfaces. We have successfully implemented electromagnetic feet with soft toes (EROFT) on an inchworm-inspired pipe crawling robot for adaptive, versatile, and stable locomotion. Foot-to-surface adaptability is provided by the inherent elasticity of the soft toe, making the robot a versatile and stable metal pipe crawler. Experiments show that the robot crawling success rate reaches 100% on large diameter metal pipes. The proposed hybrid rigid-soft feet (i.e., electromagnetic feet with soft toes) can solve the problem of continuous surface adaptation for the robot in a stable and efficient manner, irrespective of the surface curvature, without the need to manually change the robot feet for specific surfaces. To this end, the foot development enables the robot to meet a set of deployment requirements on large oil and gas pipelines for potential use in inspecting various faults and leakages.

## 1 Introduction

**FIGURE 1:**
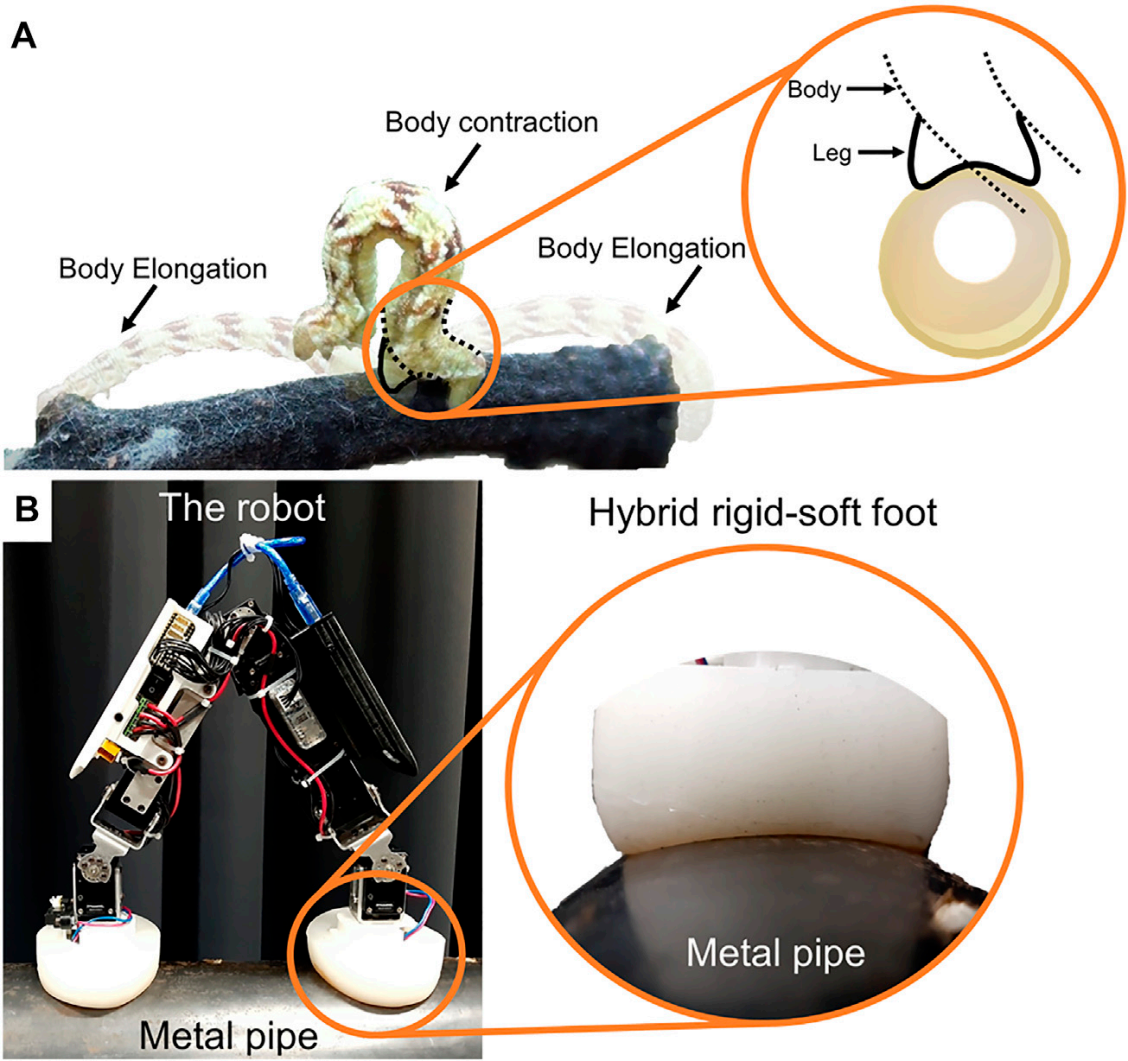
**(A)** An inchworm crawls by attaching its legs to a tree branch. The inchworm body contracts and elongates during the crawling step (looping movement). The schematic shows its legs appearing to form a concave curve while adapting to a round convex surface. **(B)** Inchworm-inspired crawling robot (iCrawl) on a metal pipe with a closeup of its hybrid rigid-soft foot completely adhering to the outer pipe surface.

Inchworms are biological creatures with an excellent ability to crawl on complex surfaces using a looping movement (Wang et al., 2014; Moreira et al., 2018) (Figure 1A), accompanied by the strong grip of its legs [9]. Consequently, inchworms can attach to complex surfaces while crawling through a highly cluttered environment with various (convex) curvatures (Figure 1A). This has inspired the development of various robots that mimic similar locomotion behavior, under various assumptions from the application perspective (Lim et al., 2008; Koh and Cho 2012; Wang et al., 2014; Moreira et al., 2018; Joyee and Pan, 2019; Yang et al., 2019; Cao et al., 2020; Niu et al., 2020).

Pipe inspection at an industrial site to assist the site-inspection operators is one key area of application (Ogai and Bhattacharya 2018). Lately, a great interest has been shown by the research and industrial organizations to tackle the remote inspection of complex sites such as oil pipelines, ships and ports, airports, renewable energy infrastructure (turbines) etc. (Lim et al., 2008; Ogai and Bhattacharya 2018; Popek et al., 2018; Ruggiero et al., 2018; Yamamoto et al., 2018). This resulted in huge institutional investments in the research and development of inspection robots. Previous works for pipe inspection developed robots with various levels of locomotion capability mainly targeting in-pipe crawling (Neubauer 1993; Lim et al., 2008; Ogai and Bhattacharya 2018). However, little attention has been given to developing robots that can perform locomotion outside the pipe (despite the advantages such as effective gas/oil leakage detection outside the pipe (Khan et al., 2020)). This is due to the nature of the outer pipe surface which involves complex structures including curvatures of various diameters from small to infinite (plate), making it a challenging problem in robot locomotion. Due to the limited scope of existing solutions for pipe gripping and adhesion (typically targeting a specific pipe diameter to grip/adhere, unlike the real-world scenarios), a significant contribution is needed to develop a functional outer pipe crawling robot for adaptively crawling on pipes of various diameters. From this perspective, learning from an inchworm’s morphology and crawling behaviors in detail can advance robot structure and locomotion control design for pipe inspection.

A functional surface attachment method is crucial for the stable movement of a pipe crawling robot. Different methods can be used to enable a pipe crawling robot to form surface attachment. The literature reveals that pipe crawling robots can use suction force, gripping force, or magnetic force to attach their legs to the surface during locomotion (Chu et al., 2010; Silva and Machado 2010). In the suction force-based method, vacuum cups are placed under the robot feet to prevent loss of adhesion due to the surface curvature (Ward et al., 2014; Kanada et al., 2019). The vacuum in the cup can be created using a vacuum pump. However, creating a sufficient adhesion force is time-consuming, and hence, may be a drawback when the aim is to achieve robust and fast locomotion. The fixed design of the suction cup could also have potential implications. Despite being somewhat adaptive, it may not adhere to the surface if there are gaps. Using a vacuum pump is also impractical since it cannot be embedded into the robot to make it fully mobile. Another method involves gripping to the surface. Mechanical grippers provide versatility such that a robot can negotiate irregular, or wire mesh surfaces (Silva and Machado 2010). While these gripper-based robots can deal with climbing surfaces to some extent, they typically exhibit limitations due to the opening constraints of the gripper (e.g., when gripping a curved surface with a curvature larger than the gripper opening). An alternative approach uses the magnetic force-based method of magnetic adhesion (Joyee and Pan 2019; Niu et al., 2020). It is inherently reliable, and hence, can effectively support robot locomotion on metal pipes (Silva and Machado 2010). Some works show permanent magnets being used in combination with wheels/tracks with no magnetic switching energy costs, while others utilize electromagnets (Chen et al., 2006; Ward et al., 2014) as the ground-contact point for the feet. While the effectiveness of magnetic adhesion is widely utilized in various robots, to the best of our knowledge, no existing work combines its advantages with a suitable robot-foot design such that the foot can adapt to various curved pipes leveraging on the magnetic adhesion, without changing the robot hardware for crawling different pipe diameters.

To fully reap the benefits of a magnetic adhesion method for a pipe crawling robot, the shape of the foot plays an important role. The overall foot design can contribute significantly to make the motion more adaptive to a surface such as a pipe. To our knowledge, no previous work considers the development of a fully generic multi-curvature pipe adaptation mechanism as part of the robot-foot design, partly restricting most existing robots to certain environments. This paper proposes a solution to this problem through a novel hybrid rigid-soft robot-foot design. Three designs are discussed in detail: minimum functional robot feet (rigid electromagnetic feet), moderately functional robot feet (rigid electromagnetic feet with rigid toes), and fully functional hybrid rigid-soft robot feet (rigid electromagnetic feet with soft toes - EROFT). We compared the proposed hybrid rigid-soft foot with the other two previously developed foot types (Khan et al., 2020). Finite element analysis (FEA) shows the new feet undergoing acceptable deformation under the given loading conditions. Other evaluation metrics include tests to examine the foot surface adaptation, foot-friction coefficient, and robot locomotion experiments with the new feet, demonstrating a significant improvement in robot performance metrics from the previously reported results.

This paper is constructed on the following main elements:

• A detailed description of how the design of the robot feet was conceived from the morphological perspective.
• Design and fabrication process of the proposed electromagnetic feet with soft toes (EROFT).
• A comprehensive investigation of various performance metrics, demonstrating the effectiveness of the proposed robot feet in increasing overall crawling success.
• Real robot experiments showing the inchworm-inspired pipe crawling robot performing adaptive, versatile, and stable locomotion by leveraging on the proposed hybrid rigid-soft robot feet.

The paper is divided into four sections: after the introduction, the robot-foot design and analysis are explained in Section 2; the experimental results are described in Section 3; and the discussion and conclusion reported in Section 4.

## 2 Materials and Methods

### 2.1 Biological Inspiration

The robot feet are inspired by the physical and behavioral attributes of an inchworm’s legs. An inchworm performs locomotion in highly complex terrains using its legs and flexible body (Wang et al., 2014). Inchworm locomotion is based on anchoring to a surface using its legs to grip, and then looping the body as it crawls. Legs on both ends of the body grip the surface while the body elongates to move in a certain direction. An overview of the inchworm movement is shown in Figure 1, where it crawls from one end of the round plant-branch by developing tension-based contraction and elongation of the body. While this movement looks relatively simple, it raises various questions, requiring a thorough understanding of the underlying morphological complexity during the crawling behavior of the inchworm. For clarity, this can be divided into two parts:

a) The morphology and movement-behavior of the flexible inchworm body.
b) The morphology and gripping behavior of the flexible inchworm-legs.

The flexible body of an inchworm is composed of soft muscle fibers. Once these longitudinal muscle fibers contract, the inchworm deforms by bending and shortening in length (Han et al., 2017; Zhang et al., 2019; Jiang et al., 2020). The inchworm-legs are foot-like adaptive organs, helping the inchworm to anchor onto a surface. Accordingly, the robot-foot design is inspired from the flexible structure and movement of the inchworm-legs and shows similar adaptation to various curved surfaces (hence, different diameter industrial metal pipes). Overall, we apply the following insights to further develop the robot feet.

1. An inchworm relies heavily on its legs to grip a surface, exerting continuous surface-compliance due to the flexible nature of the worm-body. Thus, the robot design must include a strong surface-adhesion mechanism as well as adaptive characteristics.
2. Its strong leg grip not only gives an inchworm the ability to lift its body-end but also provides movement flexibility to perform highly complex maneuvers in cluttered environments. An inchworm adjusts its movement and gripping effort depending on the environment, e.g., crawling from one tree branch to another while traversing obstacles. Thus, designing a worm-like robot requires the consideration of environmental complexity to match the task-specific needs.

We considered the above two aspects when developing an inchworm leg-inspired robot foot. The different elements of robot development are presented in the following section.

### 2.2 The Inchworm-Inspired Crawling Robot

The robot (Figure 1B) has five degrees of freedom (DOF) to perform unidirectional crawling. Each DOF is configured by a servo motor (Dynamixel XM430-W350-R). The joining assemblies are 3D printed using ABS and PLA thermoplastics. The end-to-end body length of the robot is 56 cm, with a body width of 4 cm. The robot weighs 1.53 kg, powered by a 3-cell LiPo battery (12 V, 3,000 mAh). Four sensors are mounted on the robot: a Hall sensor in each robot foot, a current sensor (ACS712, 20 A), and an infrared (IR) distance sensor at one end. The robot is an improved version of the previously developed robot reported in (Khan et al., 2020). Table 1 shows a comparison of different specifications of both versions of the robot.

**TABLE 1:** Comparison of the specifications of the two versions of the robot. Version 1 was reported in Khan et al. (2020) whereas version 2 is reported in this article.

Specification	Version 1	Version 2
Dimensions	Length = 56 cm, Width = 4 cm	Length = 58 cm, Width = 4 cm
Weight	1.42 kg (with battery onboard)	1.53 kg (without additional load)
Total DOF	5	5
Actuation type	Electrical (Dynamixel XM430-W350-R)	Electrical (Dynamixel XM430-W350-R)
Communication and controller boards	1. U2D2 (communication with computer)	1. U2D2 (communication with computer)
	2. Arduino nano (magnet drive controller)	2. Arduino nano (magnet drive controller)
Sensors	1. Dynamixel Internal encoders	1. Dynamixel Internal encoders
	2. Hall sensors at the feet	2. Hall sensors at the feet
	3. A current sensor (ACS712, 20 A)	3. A current sensor (ACS712, 20 A)
		4. An IR distance sensor
Power	A 3 cell LiPo battery (12 V, 3,000 mAh)	A 3 cell LiPo battery (12 V, 3,000 mAh)
Foot adhesion method	2 Electromagnets (5.3 W, 470 N, 12 V)	2 Electromagnets (5.3 W, 470 N, 12 V)
Additional components	Rigid toe	Soft toe and drone gripper attachment mechanism

**FIGURE 2:**
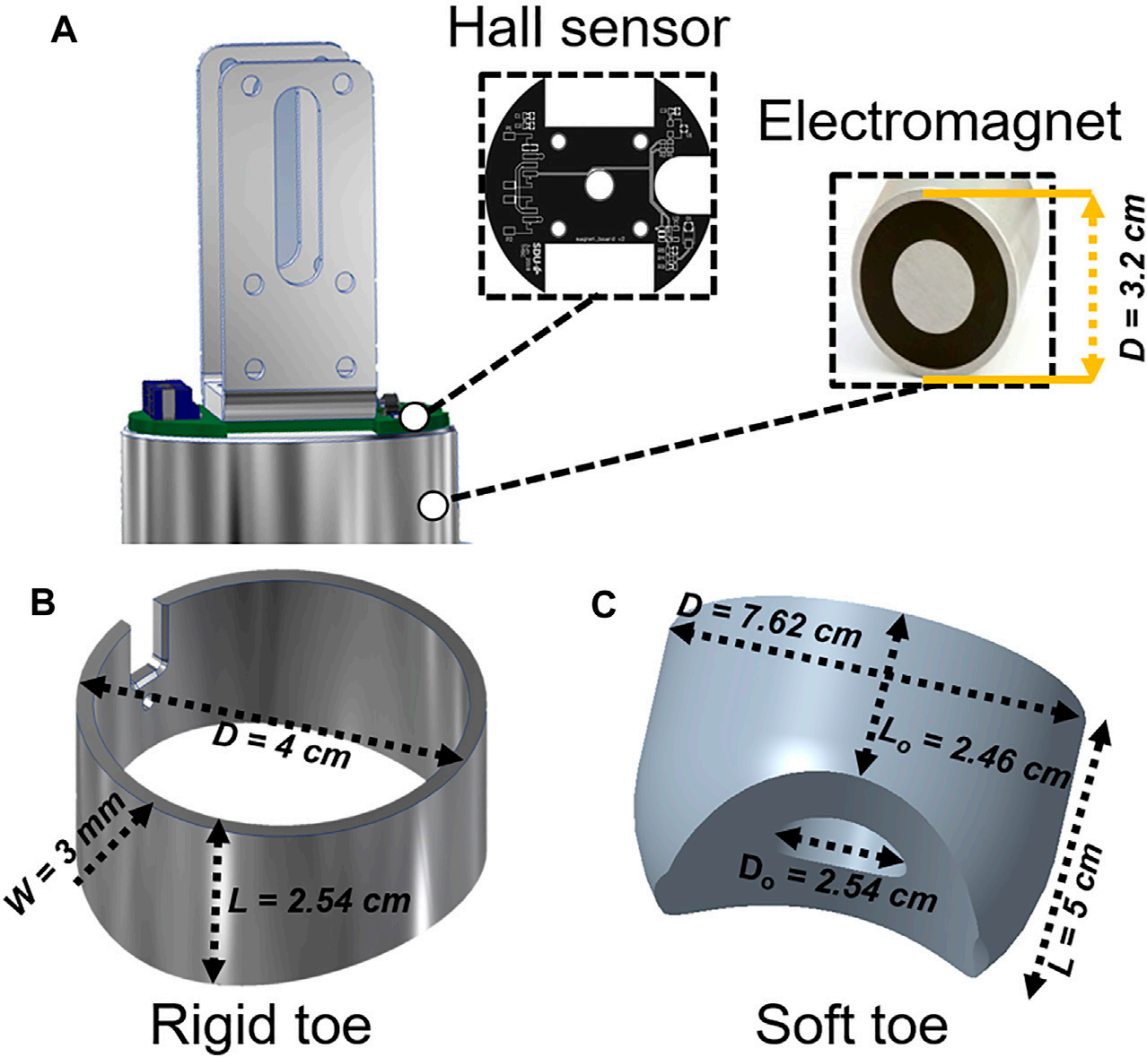
The robot has two legs: each with a foot as a metal-surface attachment mechanism. The robot feet are inspired from the functionality of an inchworm’s legs. **(A)** A computer-aided design (CAD) of the robot foot, consisting of a Hall sensor to distinguish between the metal and non-metal surfaces. The Hall sensor provides 0.78 V if the robot feet are on a metal surface, and 0.94 V when on a non-metal surface, giving the robot the capability to differentiate between the metal and non-metal surfaces. It also employs a round flux-changing permanent magnet to attach and detach the robot from metal surfaces. **(B)** A CAD of the rigid toe, mounted around the electromagnet. The toe is designed with a concave curve underneath it to passively adapt to another convex-shaped curved surface (a metal pipe in this case). It is designed to be 3D printed using PLA thermoplastics with the illustrated dimensions. **(C)** A CAD of the soft toe inspired from the structure and compliant nature of an inchworm’s legs. The soft toe could wrap the electromagnet while providing a stable attachment to all types of round surfaces. Using the illustrated dimensions (ratios, *D*_*o*_ *:D* and *L*_*o*_ *:L,* are set approximately as 1:3 and 1:2, respectively), the soft toe is designed to be fabricated with silicone elastomer following a mold casting process, as described below.

**FIGURE 3:**
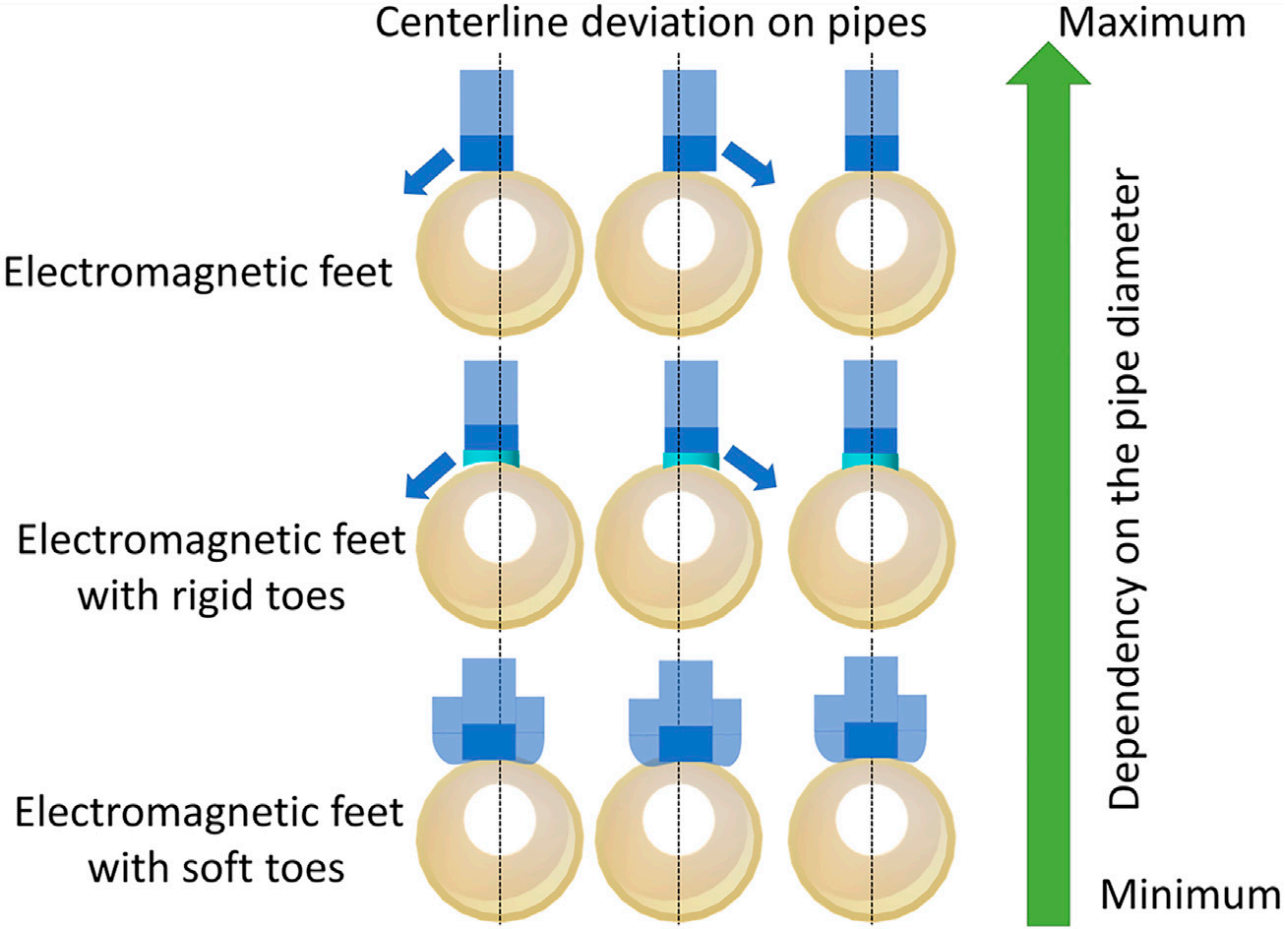
A schematic view of the robot feet and their stable movement dependency on the pipe diameter. Electromagnetic feet with soft toes can adapt easily to different pipe diameters as compared to the feet with rigid toes and flat electromagnetic feet.

The robot is designed to crawl on metal pipes of different diameters. This is the main reason we chose a magnetic adhesion method for the robot since it provides strong attachment to metal pipes. The foot consists of an electromagnet (5.3 W, 470 N, 12 V) and a Hall sensor. We call this version “the flat robot feet” (Figure 2A) since it was the first version of the robot feet which could allow the robot to crawl on flat metal surfaces. The robot feet with the curved rigid foot-toes and soft foot-toes (also here referred to as rigid toes (Figure 2B) and soft toes (Figure 2C), respectively) utilize the same hardware. However, on the metal pipes, the robot with the flat foot immediately failed to crawl since the magnets could not attach properly to the curved pipes. Figure 3 shows the robot feet deviation from a centerline on a pipe. The flat electromagnetic feet easily slip from a centerline - which defines the robot stability on a pipe. This limits the flat foot to a specific pipe diameter.

Accordingly, as the improved foot-toe design is inspired by the structure of an inchworm’s legs, a curved foot-toe (Figure 2B) design was proposed. It was 3D printed using PLA thermoplastic material. The foot-toe was mounted underneath the electromagnet such that its concave curve induced a full-face grip to the convex pipe curve. The use of the foot-toe proved to be a suitable solution (Khan et al., 2020), increasing the practicality of the earlier flat feet. However, due to its rigid nature, robot stability could only be achieved on a few of the metal pipes.

To address the issue of rigidity, we further investigated another foot-toe design with the aim of creating a versatile pipe crawling robot. Instead of plastic rigid toe, a soft toe was developed. The use of silicone elastomers is common nowadays to fabricate such a soft toe. However, recognizing the importance of suitable silicone elastomer, various elastomers were considered. Table 2 (please see at the end of the paper) provides an overview of potential choices of elastomers. Based on the FEA and the final shape of the soft toe (discussed later), Ecoflex 00-20 (SmoothOn Inc., United States) was chosen to fabricate the soft toe. Instead of being too soft or too stiff under given toe size, this elastomer avoided the restricted adaptation and enabled the foot-toe to adapt to any surface. To obtain sufficient stiffness, instead of using higher-stiffness thermoplastics with limited elastic profile (Khan et al., 2019), stiffness was achieved by ensuring the appropriate toe thickness. The process to obtain the necessary toe thickness is described next.

**TABLE 2:** Properties of silicone elastomers (Khan et al. 2019).

Type of elastomer^a^	Tensile strength (MPa)	100% modulus (MPa)	Elongation at break (%)	Cure time (hours)	Shore hardness (A⁰)	Mixed viscosity (cps)
Ecoflex 00-10	0.83	0.05	800	4	00–10	14,000
Ecoflex 00-20	1.1	0.05	845	4	00–20	3,000
Ecoflex 00-30	1.37	0.06	900	4	00–30	3,000
Ecoflex 00-50	2.17	0.08	980	3	00–50	8,000
RTV-225	≥3.4	NR^b^	≥420	2–4	28 to 39	15,000–17,000

a RTV-225 data is reproduced from seller’s shared booklet, whereas other elastomers are reported from “Smooth On” [https://www.smooth-on.com/product-line/ecoflex/.

b Not reported by the manufacturer].

**FIGURE 4:**
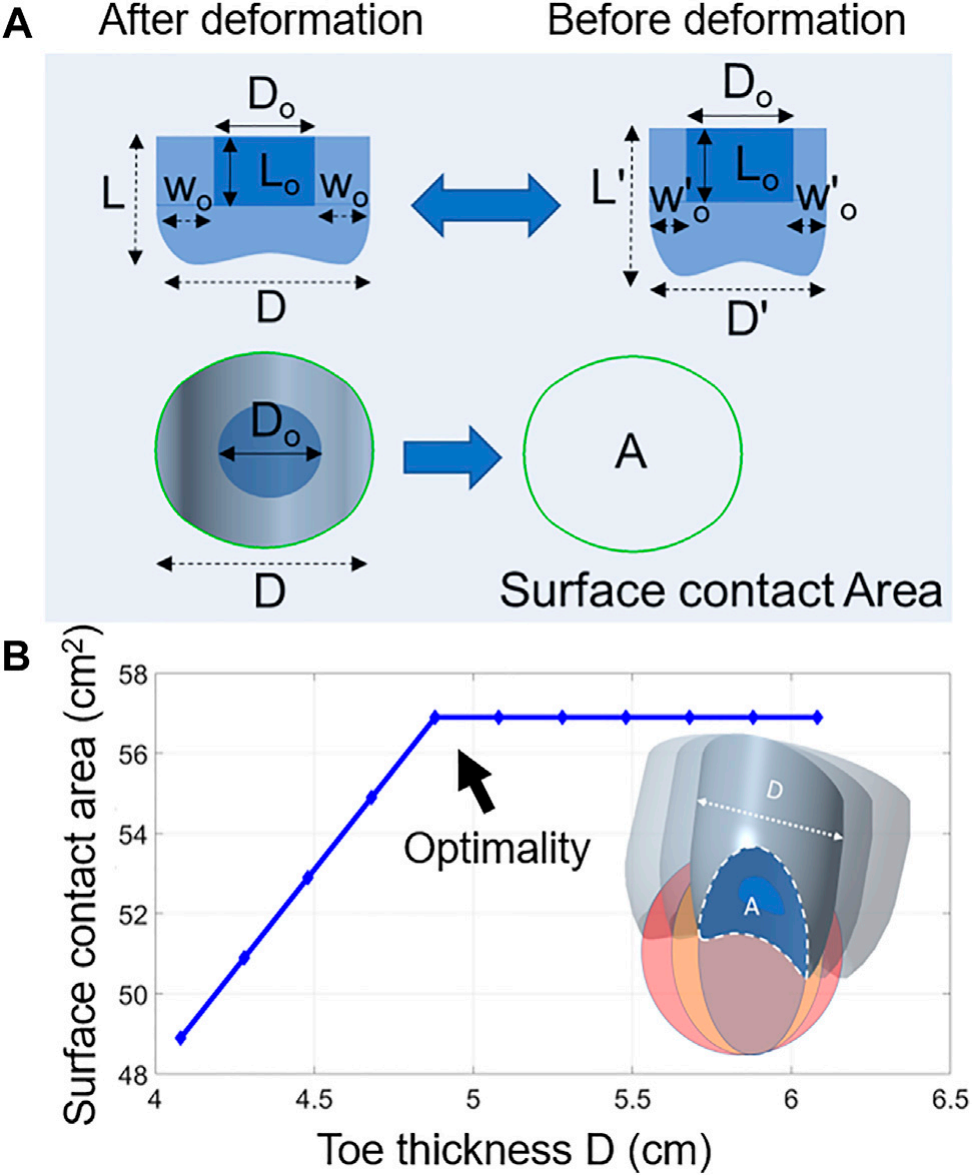
**(A)** A schematic of the soft toe after and before deformation. **(B)** A graph of toe thickness D and the surface contact area A. This was done by using Creo software to measure the soft toe’s contact area with the surface. Toes of various thicknesses were attached to the CAD of pipes. The software then calculated the real contact area between the toe and the pipe.

The toe dimensions were set following the practical design parameters. The dimensions of the toe and its cavity were based on employment of the magnet size (fixed), and the minimum diameter of the pipe on which the robot is required to crawl. For instance, if the robot crawls on a metal pipe with a diameter of 12 cm, the curved cavity must fully attach full face to the surface while inserting its maximum effective adhesion. This ensures that the robot’s feet exert the maximum grip onto a surface. Figure 4 shows the shape of soft toe in more details. In contrast to fixed-width rigid toe, the total diameter D of the soft toe changes to D′ with shrinkage of the symmetric silicone toe width w_o_ upon attachment to different sizes of pipe. The magnet dimensions are fixed (D_o_, L_o_). The outer diameter (D) adds to the required stiffness for the robot foot to stay in form. The toe width w_o_ formed by the silicone between the inner (D_o_) and outer diameter (D) was sufficient to stop undesired foot-deformation. The overall toe thickness was set such that the total toe contact surface area A is maximum. Increasing A results in increasing the elastomer’s inherent friction (see *Discussion* section).

The foot-toe was mounted around the electromagnet such that the magnet continued its exposure to the attachment surface. Meanwhile, the foot-toe prevented the robot feet from becoming imbalanced on the curved pipes. Having found these insights, we conducted FEA on both rigid and soft foot-toes, as detailed in the following section.

### 2.3 FEA Simulation

**FIGURE 5:**
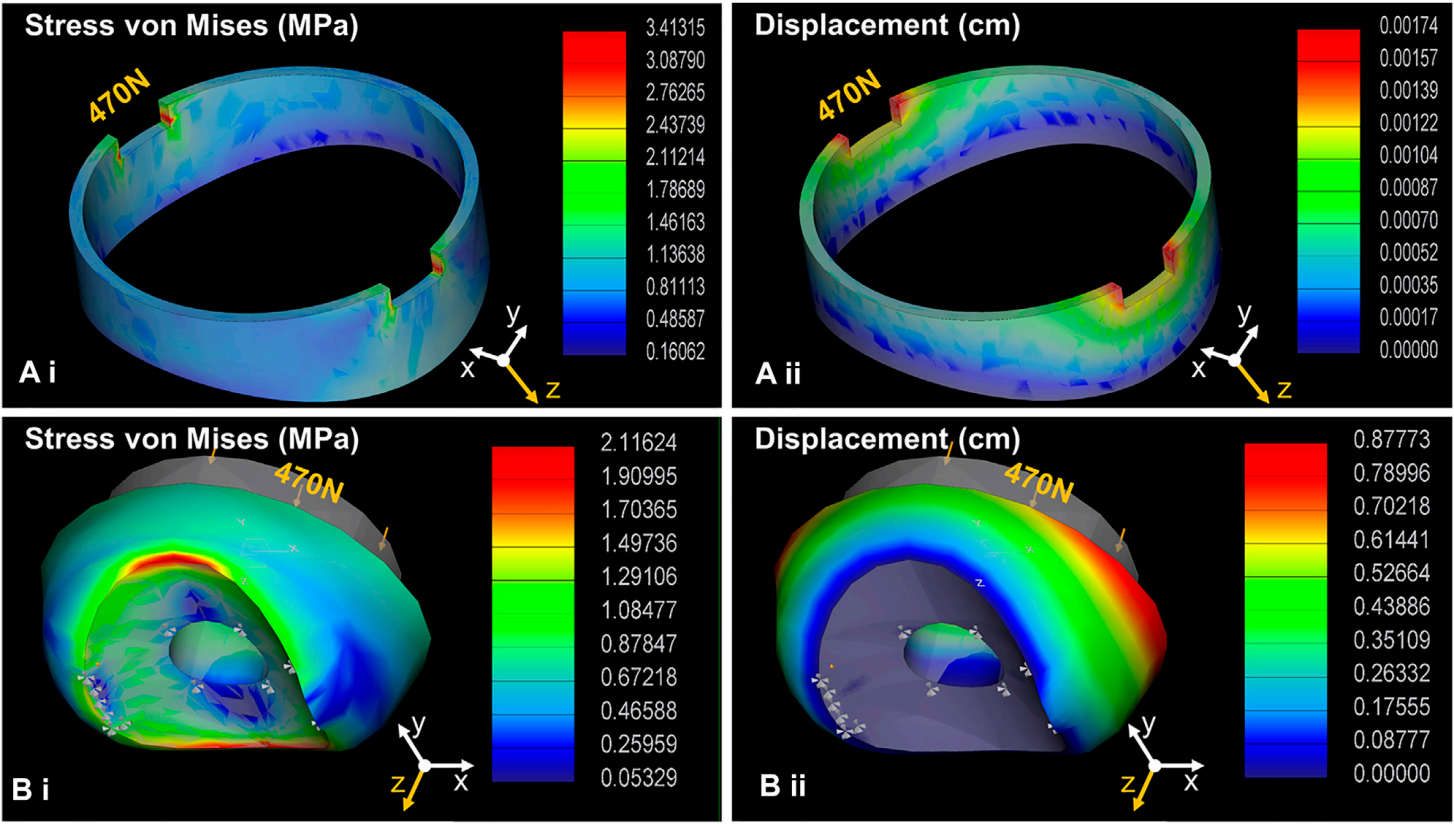
The robot-foot design relies heavily on the way it interacts with a surface. The first contact point on the surface is proposed as foot-toes along with the electromagnet. The electromagnet has a steel-case strong enough to bear the magnet’s maximum attachment force (approximately 470 N representing the force exerted by the magnet on the metal surface during attachment). To examine any deformation/displacement and stress on the foot-edge during its interaction with a metal surface, FEA simulation was performed. The simulation also allowed cross-examination of the chosen foot-toe dimensions and materials to ensure their appropriateness for compliant and safe interaction under real-life loading conditions. The simulation reveals that under the 470 N loading condition applied from the top, both foot-toes undergo an acceptable level of stress **(A.i, B.i)** and deformation **(A.ii, B.ii)**.

The robot foot lands on a surface by the toe first making contact. The stresses needed to be analyzed since the robot’s own body weight or the electromagnet could exert a strong force on the toe during magnet-attachment. The analysis would ensure that the chosen materials and geometry possessed the necessary compliance and safety under these conditions (Khan and Smithmaitrie 2018). Both the rigid and soft toe designs were analyzed under real-world-like loading conditions, based on the magnet pull up test conducted earlier (Khan et al., 2020). The maximum effective adhesion (approximately 125 N) produced by the electromagnet while fully attached to a metal surface was less than the manufacturer’s rating of 470 N. Thus, 470 N was chosen as the maximum normal force acting on the toe. Additional 100, 200 and 300 N loads were incrementally used to record the material response under the loading condition in the FEA implemented using the Creo Simulation software (Creo Simulation, PTC, United States). PLA thermoplastic material was assigned to the rigid toe using a linear model, whereas the silicone elastomer was assigned to the soft toe. A hyperplastic Mooney-Rivlin model was used to create the nonlinear properties of the material (Manti et al., 2015). The bottom of the foot-toe was constrained as a fixed point (boundary condition) to set the ground contact to properly analyze the normal force on the toe acting from the top (the load). By applying the Automatic Geometric Element Mesher (AutoGEM) of Creo software, a total of 2,398 tetrahedral mesh elements were generated for the rigid toe, whereas 6,850 elements were generated for the soft toe. The simulation results are shown in Figure 5. The maximum Mises stress of 3.41315 and 2.11624 MPa, and the maximum displacement of 0.00174 and 0.87773 cm were obtained for the rigid and soft toes, respectively. Table 3 (please see at the end of the paper) summarizes the FEA under 100, 200, 300, and 470 N loading conditions. The detailed simulated FEA results are shown in Supplementary Figures 1 and 2.

**TABLE 3:** A comparative overview of FEA results for the rigid and the soft toe.

FEA result	Loading condition (N)	Rigid toe	Soft toe
Minimum total displacement (cm)	100	0	0
	200	0	0
	300	0	0
	470	0	0
Maximum total displacement (cm)	100	3.703e-04	0.18675
	200	7.405e-04	0.37350
	300	0.00111	0.56025
	470	0.00174	0.87773
Minimum Von Misses stress (MPa)	100	0.03417	0.01134
	200	0.06835	0.02268
	300	0.10252	0.03402
	470	0.16062	0.05329
Maximum Von Misses stress (MPa)	100	0.72620	0.45026
	200	1.45241	0.90053
	300	2.17861	1.35079
	470	3.41315	2.11624

This demonstrates that the overall deformation and stress for both foot-toe designs were within the safe limit. Considering this initial assessment, toe fabrication was initiated, as detailed in the following section.

### 2.4 Foot-Toe Fabrication Process

**FIGURE 6:**
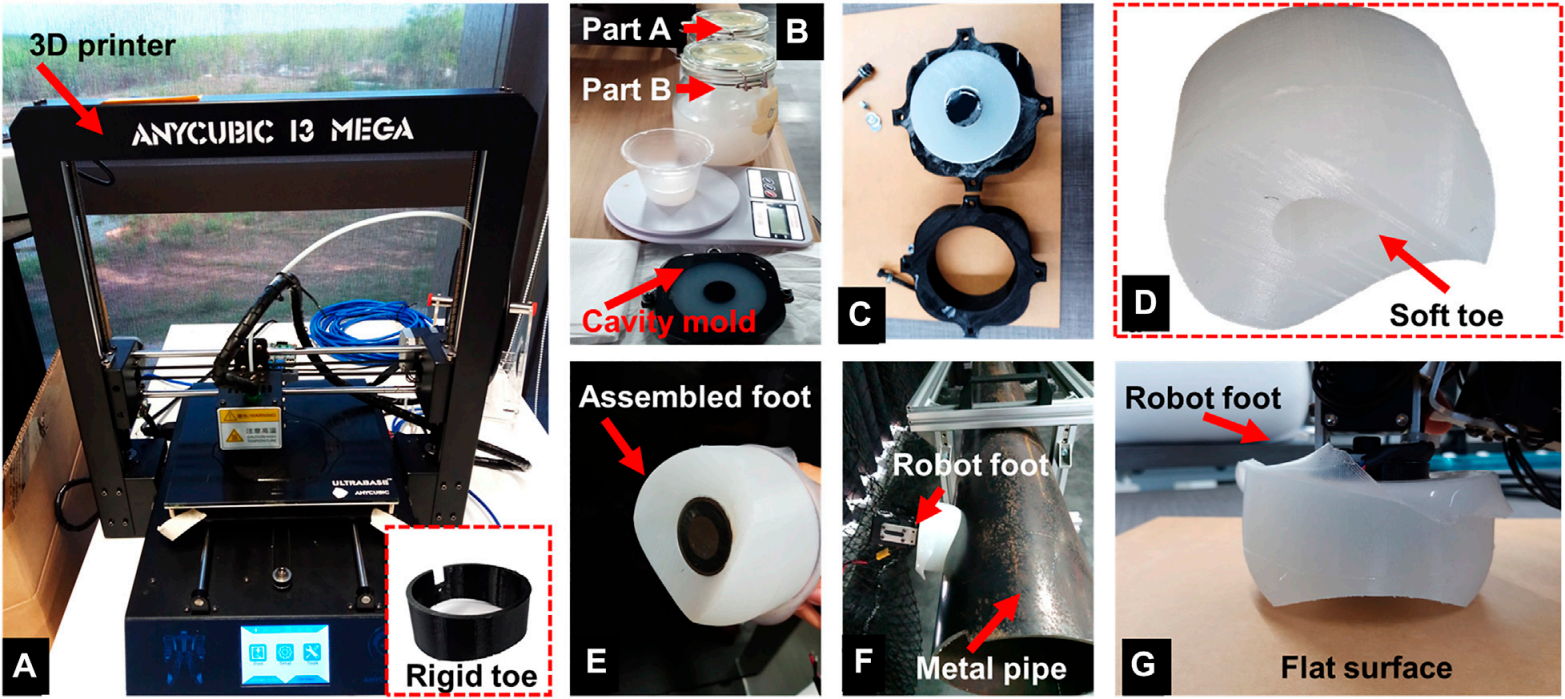
An overview of the foot-toe fabrication process. **(A)** A 3D printer was used to fabricate the PLA based rigid toe (shown in the bottom inset), and a cavity mold for the silicone-based soft toe. **(B)** The silicone elastomer (parts A and B) was mixed in a weight ratio of 1:1 (by weight) and poured into the cavity mold. After mounting the negative part on top, the mold was left for about 4 hours at room temperature to cure. **(C)** Post curing, the negative part was unmounted resulting in the soft toe D). **(E)** The soft toe was attached to the rigid frame with the electromagnet such that the magnet could fully face it underneath. **(F)** The hybrid rigid-soft foot on the metal pipe with the soft toe adapting to the pipe diameter. **(G)** The hybrid rigid-soft foot on a flat surface with its edges undergoing minor deformation while the magnet remains fully attached.

A 3D printer was used to fabricate a rigid toe weighing 7 g using PLA thermoplastic material (Figure 6).

To fabricate the soft toe, a cavity mold was produced using a 3D printer, consisting of two parts: cavity and negative. Ecoflex 00-20 elastomer parts A and B were mixed and poured into the cavity mold. The mixture was kept at room temperature for 4 hours to form the elastomeric toe by decoupling the cavity mold from the negative part. The toe weighed 156 g. The rigid foot-toe was mounted on the robot foot under the magnet and fixed with screws. The magnet and inner diameter of the rigid toe were matched for tighter toe-grip. To mount the soft toe, the electromagnet was placed inside its cylindrical cavity, tightly gripping the electromagnet without any slippage during the attachment/detachment process. This was designed such that the magnet diameter was roughly 1.5 times that of the inner toe-cavity, resulting in a strong grip. The hybrid rigid-soft foot is a combination of the rigid frame with the electromagnet and the soft toe. It was fully compliant on flat and round metal surfaces. Figure 6 illustrates the fabrication of the foot-toes. After fabrication, the robot-foot designs were tested and compared in the various experiments described in Section 3.

## 3 Experiments and Results

In this study, we performed four main experiments to investigate the performance of the hybrid rigid-soft foot and compare it with other types. Each experiment and its results are described below.

### 3.1 Adaptability Experiments

**FIGURE 7:**
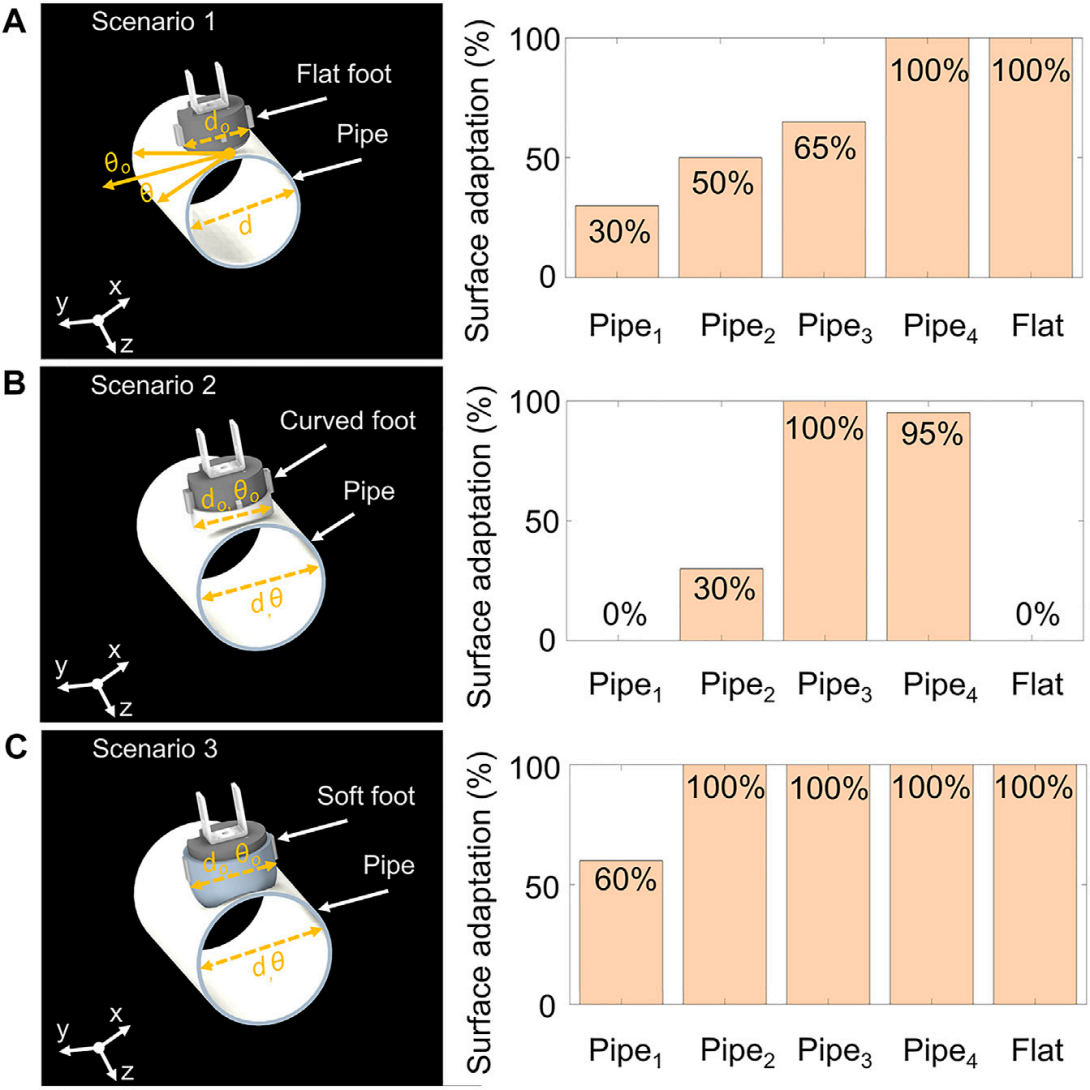
An experiment to compare and analyze the percentage foot-adaptation on different metal surfaces when the bottom of the robot foot was flat, curved with the rigid toe, and the soft toe. This experiment was used to consider instances in which the robot failed a crawling experiment solely because of mismatching feet and a curvature in the crawling surface. **(A)** The first scenario shows that the flat robot foot (*diameter* = *d*_*o*_) adhered to a metal pipe (*diameter* = *d*), where the angle formed between the foot face (shown as *θ*_o_) and pipe (shown as *θ*) contributed to foot-adaptation when fully attached to a metal pipe. Here the pipes_1,2,3,4_ have diameters of 12 (minimum case), 16 (medium case), 22 (practical case), and 60 cm (potential case), respectively. **(B)** The second scenario utilized the curved rigid foot-toe, and its surface adaptation demonstrated on all test surfaces. **(C)** The third scenario employed the soft curved foot-toe, and the resulting surface adaptation can be observed.

This test compared the foot designs with all the test surfaces based on their adaptation level. The surface adaptation metric was proposed, and calculated according to the percentage of foot-bottom area fully attaching to a surface. Figure 7 shows the three scenarios and their results.

The first scenario (Figure 7A) shows the flat foot tested on multiple pipes and a flat surface. The second scenario (Figure 7B) shows the curved rigid foot, while the third scenario (Figure 7C) shows the hybrid rigid-soft foot. The angles (θo,θ) formed by joining the robot feet (diameter = do) and pipe (diameter = d) were noted by recording images on a camera placed in parallel to the pipe. These images were processed to obtain the angles formed between the robot feet and pipes from a physically marked reference point. The summation (Σθ = θo + θ) from a reference point was normalized as the percentage foot-to-surface adaptation. For example, if the foot and pipe were fully attached (i.e., Σθ = 0), the resulting surface adaptation was 100%. The results obtained by this test showed consistency with the crawling success rate (reported later in this paper). It showed that the foot-toe design significantly influenced robot crawling success.

### 3.2 Deformability Experiments

**FIGURE 8:**
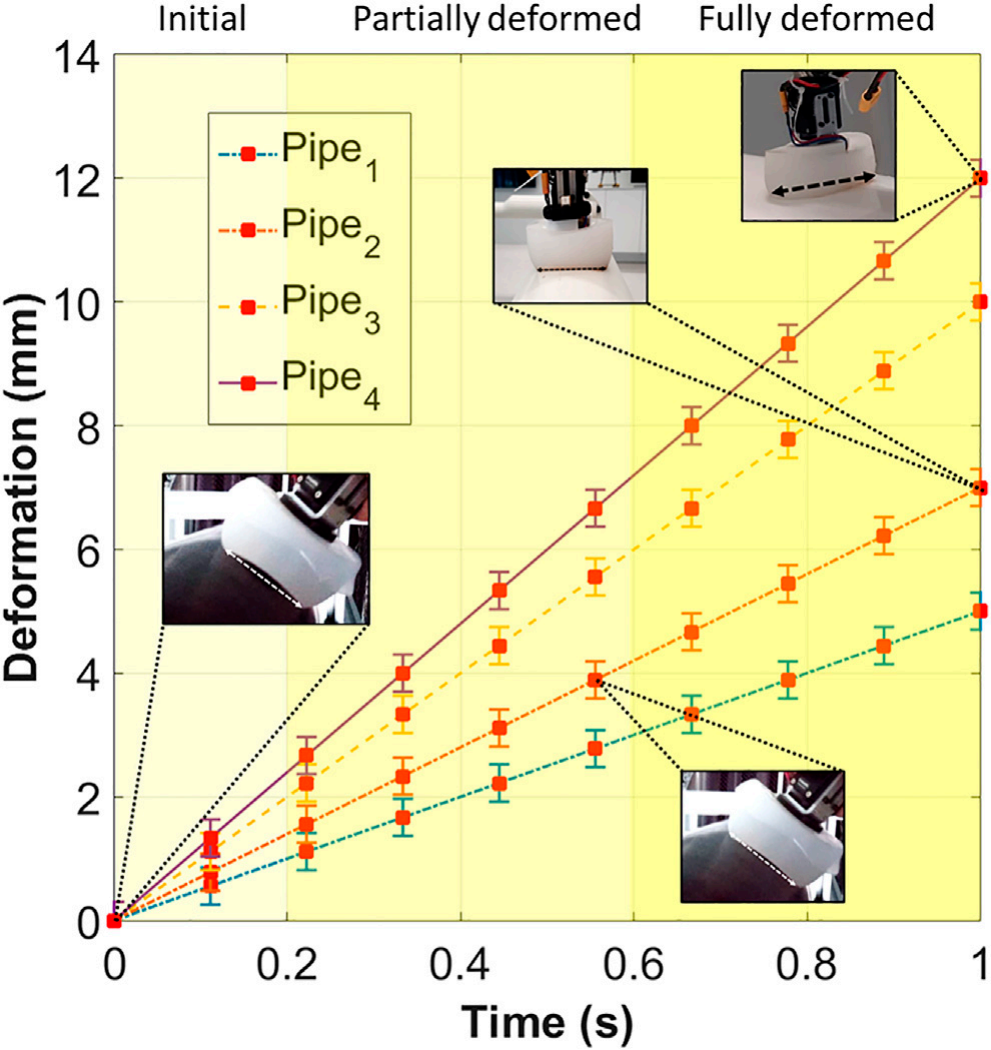
The deformation profile experiment showing the soft toe on different pipes. The soft toe adapts passively while complying with the curvature of the pipe; enabling the robot to crawl on any size of pipe in its current configuration. Here the pipes_1,2,3,4_ measure 12 (minimum case), 16 (medium case, tested on painted and non-painted pipes), 22 (practical case), and 60 cm (potential case) in diameter, respectively. The change in deformation is proportional to the curvature of a pipe. An increase in the diameter of the pipe results in a corresponding increase in the foot-toe deformation once the robot foot is attached to a metal pipe. The *y*-axis shows the deformation as displacement, while the *x*-axis shows the time stamp from a recording which is utilized to observe how the soft toe deforms to the surface.

From the FEA, we noted that the foot-toe designs could experience acceptable deformation and stress. Due to the materials used, the rigid toe may either not deform, or permanently deform if the applied load surpasses the PLA thermoplastic strength limit. However, the elastomeric soft toe underwent elastic deformation such that it could recover its original shape even after bending, shrinking, or elongating under a specific applied load condition. To further examine this specifically for the metal pipe crawling scenarios, its deformation profile was obtained. A camera recorded the foot-toe deformation (toe-curve spread) once the robot foot had attached to the metal pipe. The recording was analyzed to obtain the deformation profile as shown in Figure 8. The experiment was conducted on various pipes with diameters of 12, 16 (painted and non-painted), 22, and 60 cm. The fully recoverable foot-toe deformation showed the robot feet had the capacity to adhere to outer pipe surfaces of any diameter.

### 3.3 Friction Experiments

**FIGURE 9:**
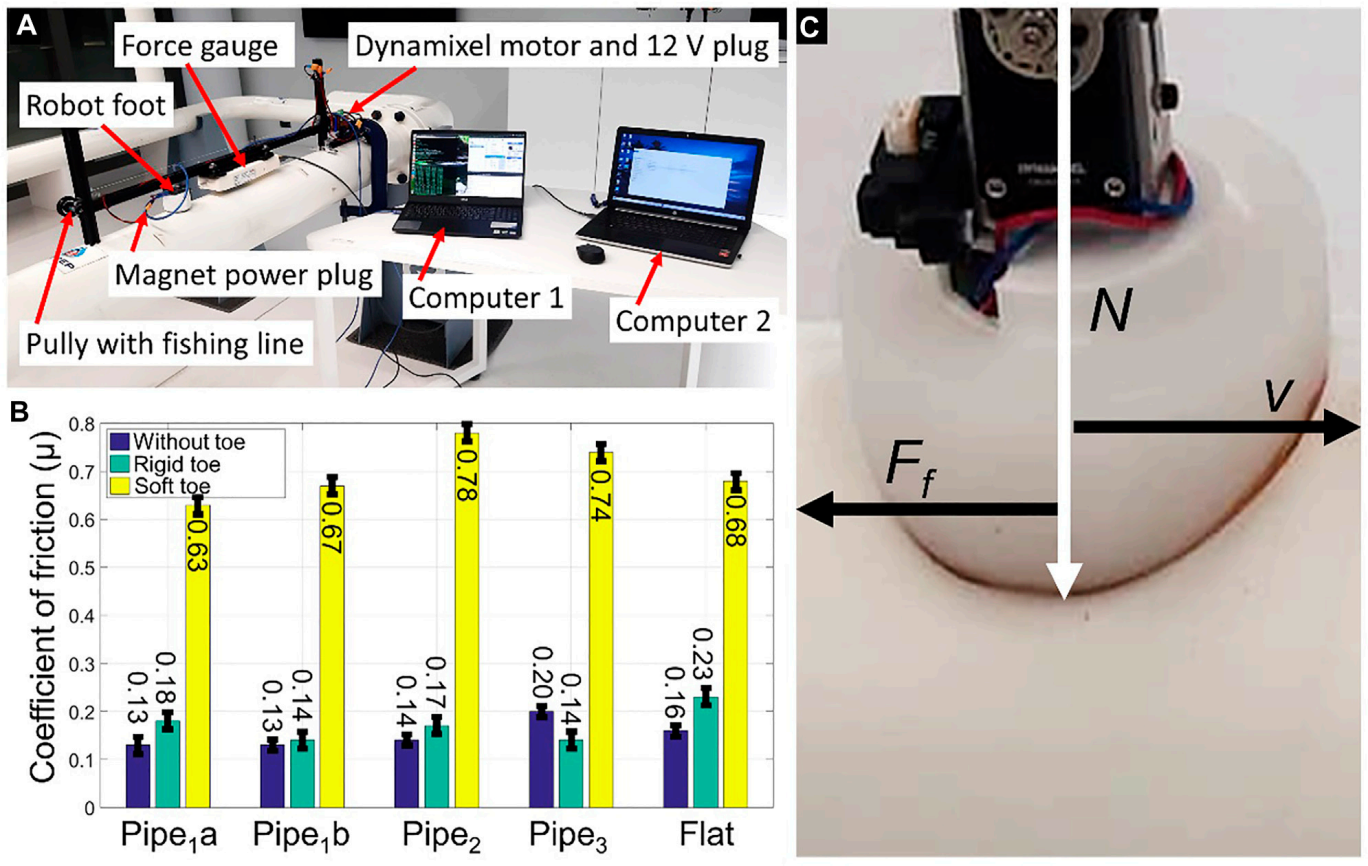
**(A)** The experimental setup for measuring the friction coefficient associated with all tested foot designs. A force gage was attached to the robot foot mounted on an aluminum frame. The robot foot was then moved along the different metal surfaces. The magnet was switched to *ON* to avoid the magnetic force impacting the measurements. Two separate computers controlled the movement of the Dynamixel motor and recorded the force data, respectively. **(B)** The friction coefficient of the robot foot under various test conditions. Pipes_1_a,b represent the metal pipes without and with painted surfaces, each with a diameter of 16 cm. Pipes_2,3_ measured 22 and 60 cm in diameter, respectively. The tests were repeated 10 times for each tested surface and all robot-foot designs. **(C)** The robot feet pushing on the metal pipe with normal load N resulting in a friction force F_f_, while stroking it with velocity v.

The difference in friction between any two surfaces plays an important role in understanding their interaction. The friction coefficient is used to assess the resistance between the two adjacently sliding surfaces (Wang et al., 2014; Manoonpong et al., 2016). The robot foot was tested to obtain the friction coefficient for different metal pipes and a flat metal plate. The experimental setup to obtain friction coefficient of all toe designs and the results are shown in Figure 9. The soft toe was found to increase the friction coefficient in comparison to the flat or rigid curved foot due to the maximum adjacent foot-sliding area. This helps the robot to avoid slipping on painted metal surfaces during crawling.

### 3.4 Robot Locomotion Experiments

**FIGURE 10:**
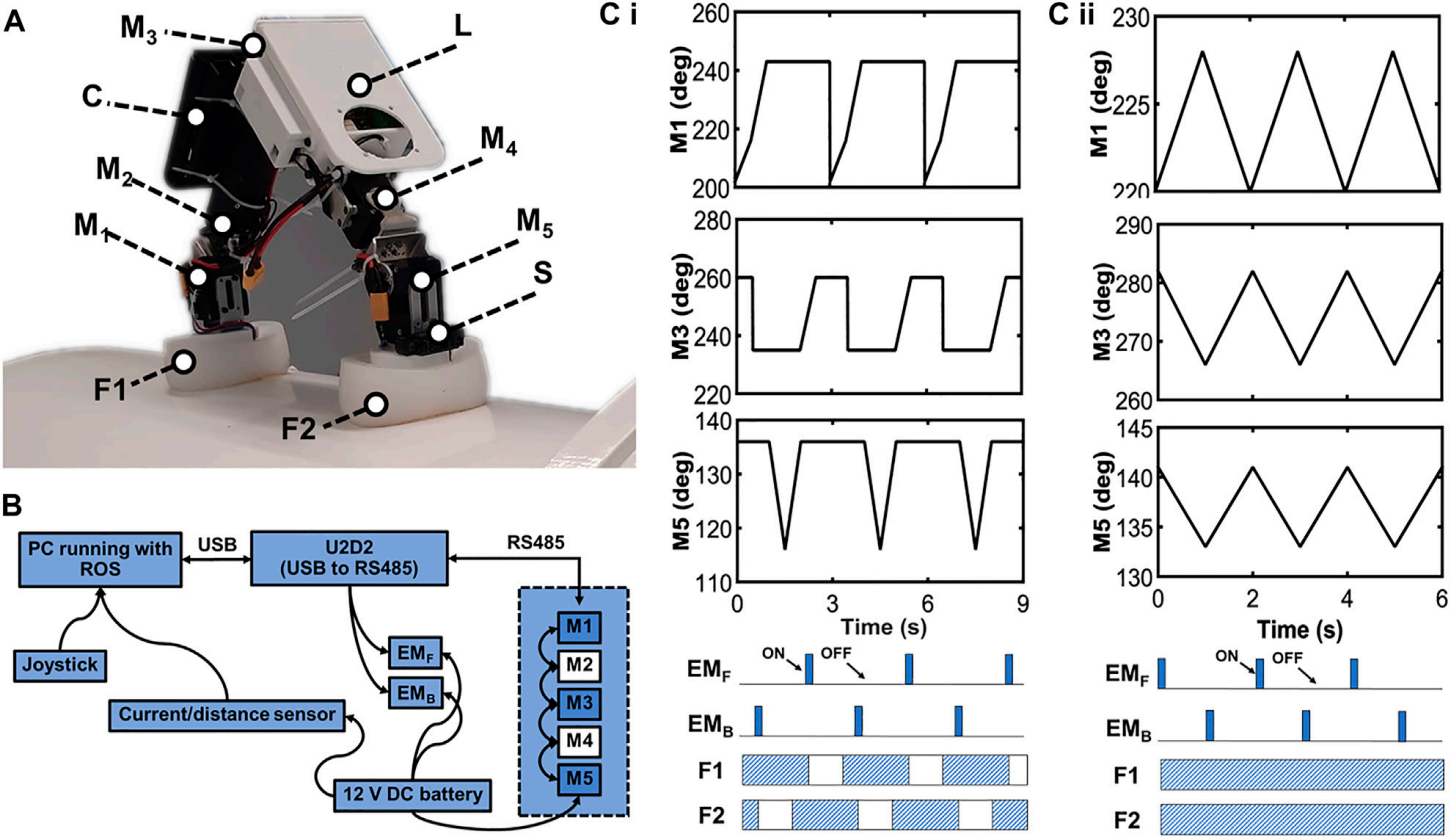
An overview of the inchworm-inspired crawling robot. **(A)** The labeled photograph of the robot standing on a metal pipe. Here, M_1,2,3,4,5_ denote the five servo motors; S is the IR distance sensor; C is the control board holder; L is the additional payload space, or alternatively a lifting mechanism, and F_1,2_ are the rigid-soft robot feet. **(B)** The robot system diagram. The M_2,4_ servo motors were kept locked to allow the other motors to be controlled for unidirectional robot movement. **(C,i)** The robot motor signals for the unidirectional *stepping gait*, magnet switching diagram, and resulting gait pattern. **(C,ii)** The robot motor signals for the unidirectional *sliding gait*, magnet switching diagram, and resulting gait pattern. Gait generation is achieved by state-machine control (see Khan et al. (2020) for details).

According to the results of the previous experiments, the proposed hybrid rigid-soft foot outperforms the other two types. Therefore, we used it to further test robot locomotion. The robot performed unidirectional crawling by utilizing two gaits: the stepping gait where the robot lifts its body weight on one of its legs, and the sliding gait where the robot stays inbound to the ground and performs a sliding-like motion (Khan et al., 2020). The robot motor joints M1,3,5 were configured to move, with M2,4 kept locked at an angle for unidirectional crawling. The magnets’ alternating switching pattern follows the gait design. The overall robot hardware and system components, motor signals, magnet switching, and gait patterns are illustrated in Figure 10. Details of the robot locomotion control are not the main focus of this study and can be found in Khan et al. (2020).

**FIGURE 11:**
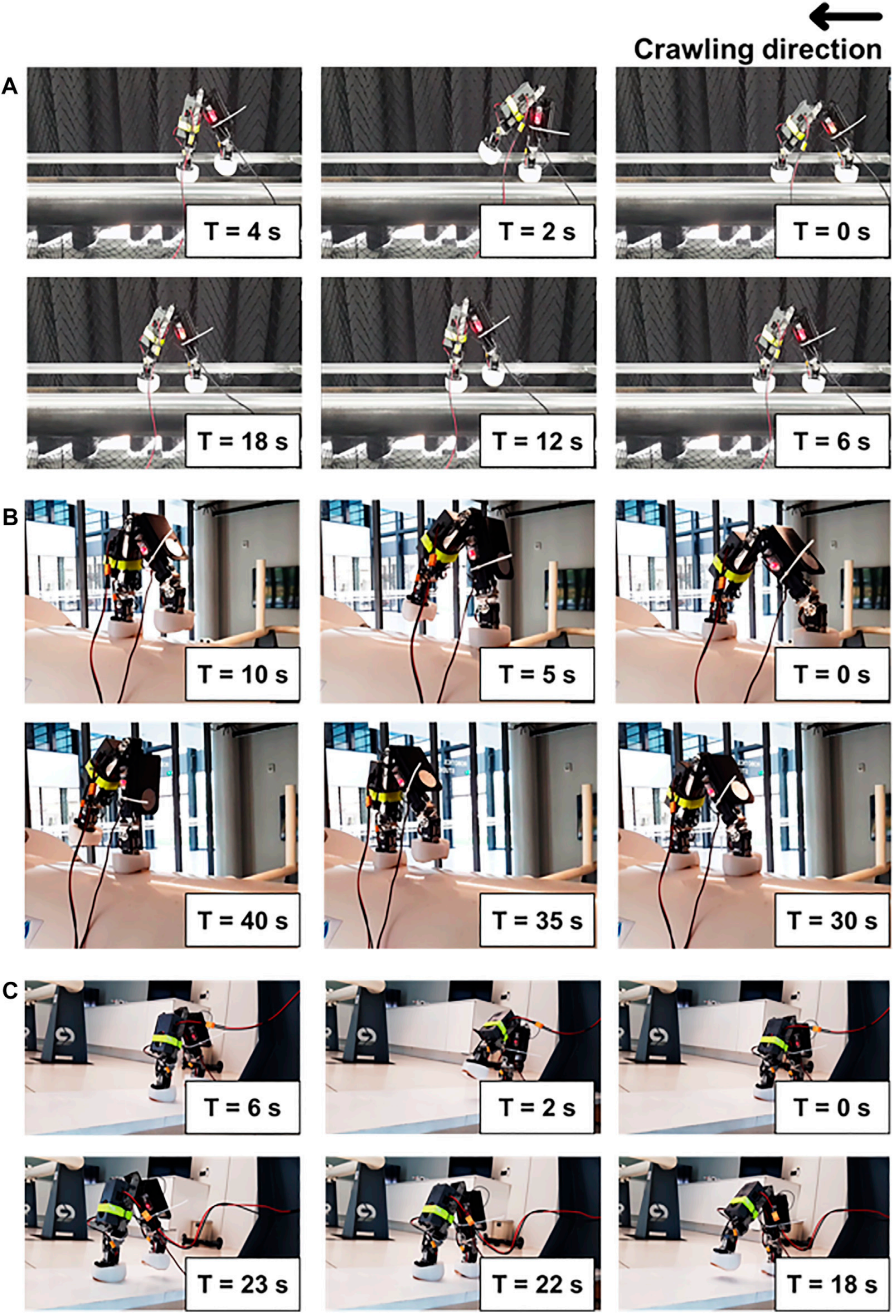
The inchworm-inspired crawling robot performing point-to-point locomotion on metal pipes: **(A)** pipe diameter = 16 cm, **(B)** pipe diameter = 60 cm, and **(C)** flat metal plate, using the hybrid rigid-soft foot and *stepping gait*. The robot crawling speed with the stepping gait was 8.9 mm/s. Using the sliding gait, the robot speed was 25 mm/s. A supplementary video of the experiments can be viewed at http://www.manoonpong.com/EROFT/videoS1.mp4.

Utilizing gaits 1 and 2, the robot performed point-to-point crawling on metal pipes and a flat metal plate. The robot crawling speeds with gaits 1 and 2 were 8.9 and 25 mm/s, respectively. Other representative crawling experiments are presented in Figure 11. Figure 11A shows the robot crawling on a medium-sized pipe (diameter = 16 cm) using gait 1 and the hybrid rigid-soft foot. Another experiment on a very large pipe using gait 1 and the rigid-soft foot is shown in Figure 11B. The robot crawling with the hybrid rigid-soft foot on the flat metal plate is shown in Figure 11C. Robot locomotion behaviors using the sliding gait can be viewed at http://www.manoonpong.com/EROFT/videoS1.mp4.

**FIGURE 12:**
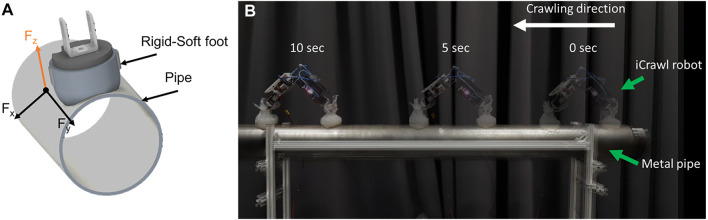
**(A)** Rigid-soft foot leverages on the adhesion and friction components (F_x,y_) upon attachment to the metal pipe. **(B)** The robot performs high-speed bidirectional crawling on a metal pipe with a speed of 100 mm/s using the rigid-soft feet. A supplementary video of the experiments can be viewed at http://www.manoonpong.com/EROFT/videoS1.mp4.

A high-speed locomotion test showed that the robot can perform highly robust and stable locomotion on industrial pipes. Figure 12 shows an overview of the experiment. The robot leveraged on the friction and adhesion force components added by the soft toe, increasing the stability of the robot. It performed bidirectional crawling without any failure on industrial pipes with a speed of 100 mm/s – surpassing its previous maximum speed of 25 mm/s (Khan et al., 2020), while outperforming existing similar robots on this metric (refer to the speed column in Figure 14).

**FIGURE 13:**
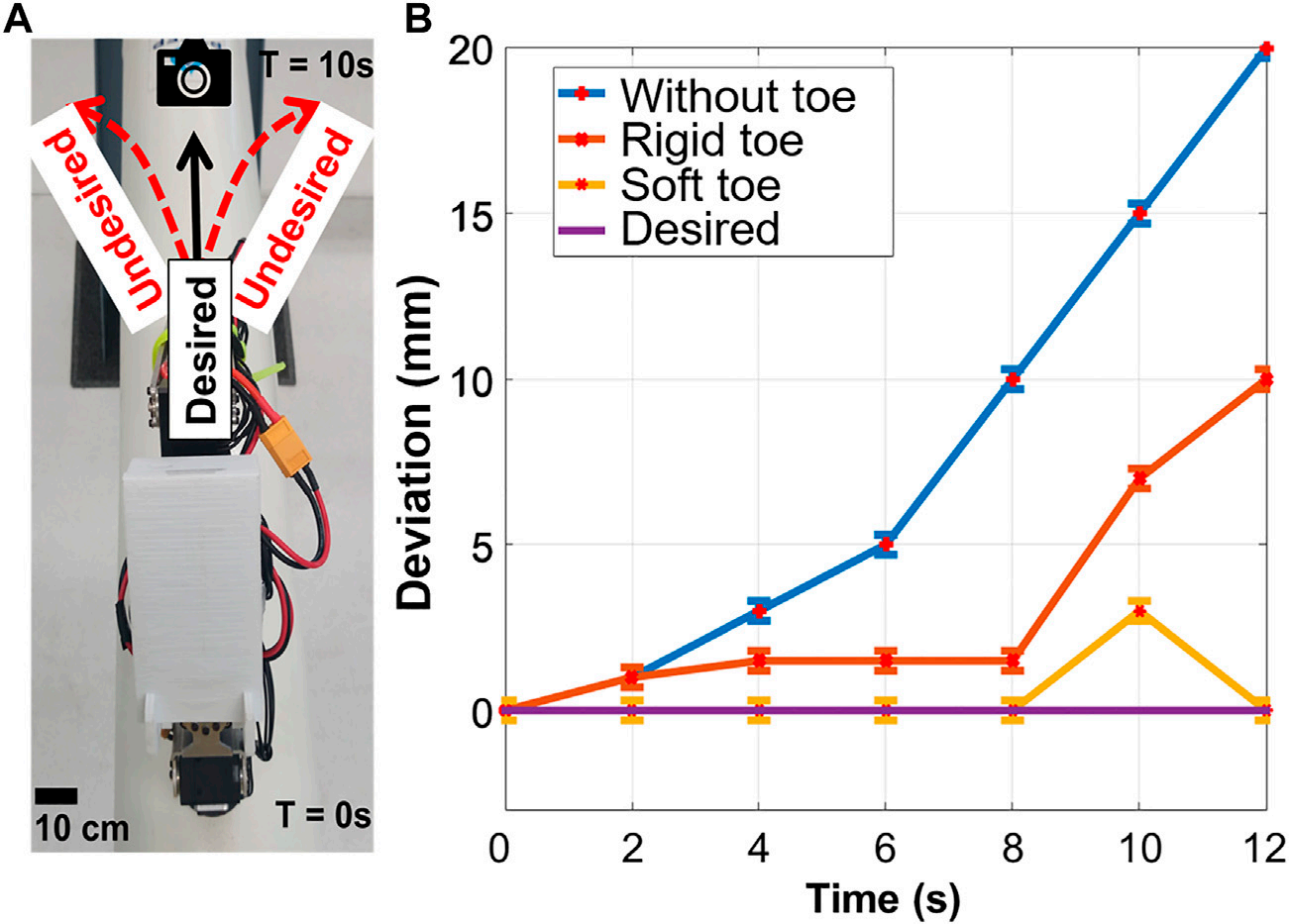
**(A)** Top view of the robot and **(B)** the distance in its deviation profile with respect to the desired crawling path. The robot performed point-to-point locomotion with minimum deviation from its unidirectional crawling path using the soft toe. The deviation was found to be consistent on all pipes.

Earlier in the Figure 3, robot feet were compared for their deviation profiles. To obtain actual deviation profile of the robot from the desired path during locomotion, a top-view camera was used to obtain its actual (Figure 13). This was to observe the change in undesired robot-body oscillation due to the different foot-toes employed, resulting in any robot deviating from a path. The robot was found to benefit from reduced perturbations and foot-slip-based failure due to the compliant toe design during attachment and detachment of the magnetic feet.

Using the hybrid rigid-soft feet, the robot crawled on all surfaces with an improved success rate. The crawling success rate was based on the point-to-point locomotion of the robot for each foot type, the employed gait, and type of surface. Table 4 (please see at the end of the paper) summarizes the robot crawling success rates for all robot locomotion scenarios. Experiments were repeated five times for each scenario. The key insights arising from the experimental results are discussed in the next section.

**TABLE 4:** Robot crawling success rate for various metal surfaces under different conditions.

Robot-foot type	Gait type	Success rate (%) w.r.t the surface type				
		*Flat*_*plate*_	*Pipe*_*1*_	*Pipe*_*2*_	*Pipe*_*3*_	*Pipe*_*4*_
Electromagnetic feet	Gait 1	100	10	10	10	100
	Gait 2	100	75	80	80	100
Electromagnetic feet with rigid toes	Gait 1	0	10	25	20	0
	Gait 2	0	95	100	100	0
Electromagnetic feet with soft toes (EROFT)	Gait 1	**100**	10	**100**	**100**	**100**
	Gait 2	**100**	75	**100**	**100**	**100**

*Note:* Flat and curved foot results are reported from Khan et al. (2020) for comparison. All the experiments used an identical setup, each repeated five times for each scenario. The bold values indicate improvement in success rate.

Based on the above experiments, it can be clearly observed that the robot-foot design played a fundamental role in robot crawling success. The flat foot was suitable only for flat surfaces, or when the pipe diameter was sufficiently large that it appeared flat to the foot. The robot foot with the rigid curved toe showed a certain level of surface conformity, increasing the robot crawling success rate on the pipes. However, under this configuration, the robot could not crawl on the flat surface or very large diameter pipes due to the rigid toe-slit restricting complete attachment, meaning it could crawl only on a few pipes. The hybrid rigid-soft foot proved to be the most effective for continuous compliance with the crawling surface, resulting in maximum success. By leveraging the hybrid rigid-soft foot, the robot also showed its capability to become a versatile pipe crawler on a medium-sized pipe with a very large diameter. The experiments for characterizing the robot feet (i.e., adaptability, deformation, friction, and deviation experiments) showed that the hybrid rigid-soft foot was superior to the flat and curved rigid foot. The hybrid rigid-soft foot deformed to all curvature pipes and retained sufficient stiffness to balance the robot. It produced high friction on all surfaces, enabling the robot to avoid critical slippage instances during crawling.

## 4 Discussion and Conclusion

Three foot designs were specifically investigated in this study, inspired from the morphology of an inchworm’s legs for a pipe crawling robot. Following the performance criteria, a new foot design for an inchworm-inspired pipe crawling robot was introduced. The hybrid rigid-soft foot was designed to create a versatile pipe crawling robot. We discussed the hybrid rigid-soft foot nature considering the criteria behind its shape, choice of materials, testing conditions, and robust integration with the robot. Furthermore, the foot designs were tested to analyze and compare their deformation and stress behavior under different real-life loading conditions. The hybrid rigid-soft foot showed maximum compliance with most tested pipes.

The foot-friction test revealed that the robot can obtain superior friction, essentially making it a highly improved version in comparison to the other two foot types. It is established by several tribological studies that a compliant structure undergoes shape adaptation which increases its real contact area (RCA) to an attaching surface (Frank et al., 1986; Duvefelt 2016; Sahli et al., 2018). Hence, an increase in RCA enhances a compliant structure’s friction under the same load as compared to a rigid structure. As a result, the gripping force of a compliant structure is also increased. The impact of RCA based on a compliance model differs from that of a traditional contact model, which is unaffected by apparent contact area (ACA). The elastomeric foot-toe, with its analysis-based choice of geometry, follows the RCA principle and creates strong RCA due to its unique compliancy and contact behavior, resulting in more friction for a stronger grip. The use of elastomeric foot-toe resulted in a significant improvement in the multi-curvature pipe crawling of the robot. The robot crawled without failure on different highly curved metal pipes by utilizing the soft toe, magnetic adhesion, and two inchworm-inspired crawling gaits. Finally, the crawling success rate was compared in all three foot types, and the proposed hybrid rigid-soft foot was found to outperform the other two (flat foot and curved rigid foot).

**FIGURE 14:**
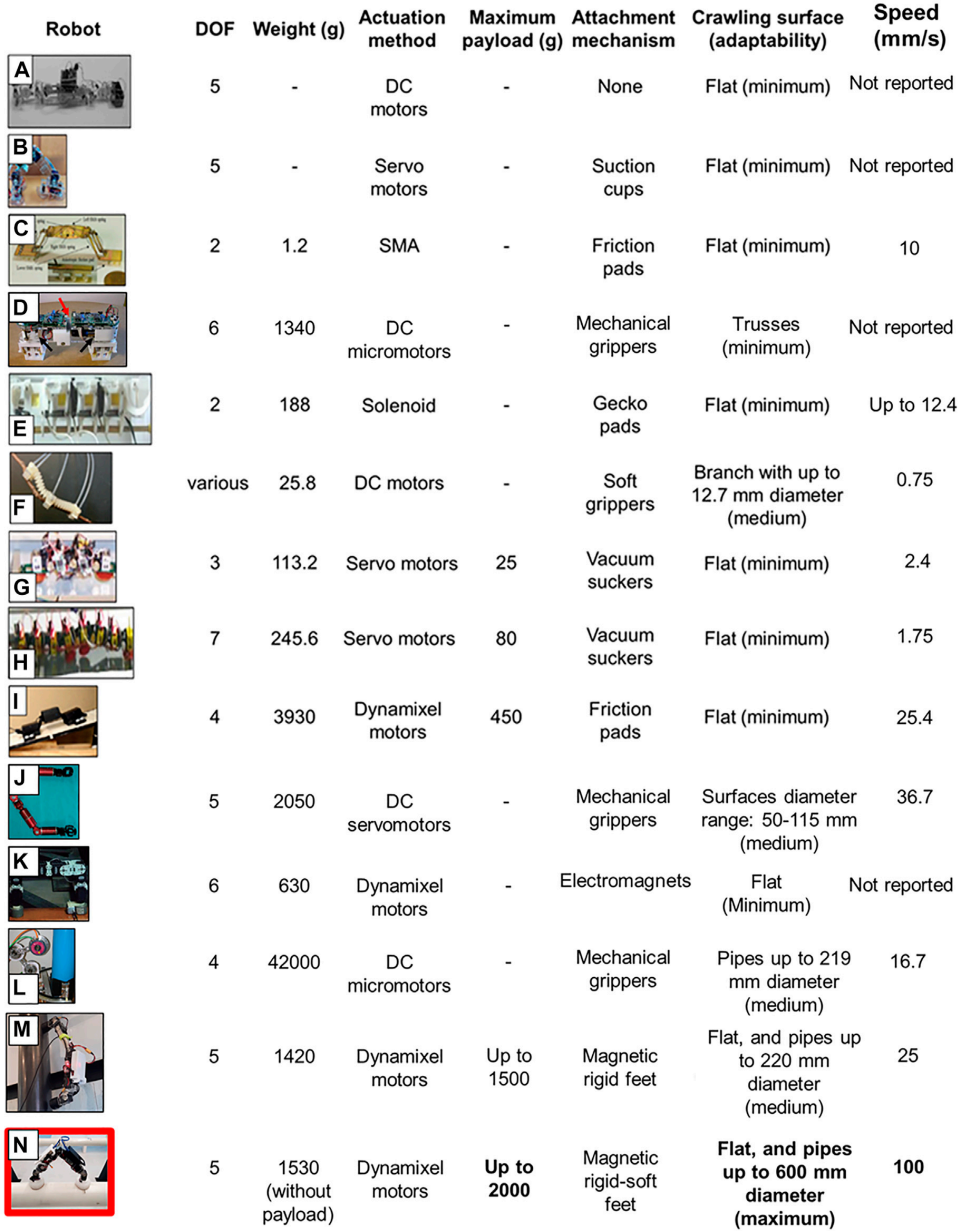
A comparison between different state-of-the-art worm-like robots **(A–L)** and our robot **(M)**. The worm-like robots are chosen for comparison based on design diversity, fabrication, and control approaches such that a broader comparative view of the worm-inspired robots can be developed. Our versatile robot relies on the rigid-soft magnetic feet to crawl with maximum adaptability on curved surfaces of up to 600 mm in diameter, carrying a comparatively superior load traveling with a higher speed. Other robots shown here are reported from the literature in the following: **(A)** reported in Avila et al (2006), **(B)** in Yuan et al. (2016), **(C)** in Koh and Cho (2012), **(D)** in Yoon and Daniela (2007), **(E)** in Han et al. (2017), **(F)** in Rozen-Levy et al. (2019), **(G)** in Wang et al. (2009), **(H)** in Wang et al. (2009), **(I)** in Moreira et al. (2018), **(J)** in Guan et al. 2016), **(K)** in Bi et al. (2012), and **(L)** in Tavakoli et al. (2008). **(M)** is the previous version of iCrawl robot reported in Khan et al. 2020), whereas **(N)** is our latest version of iCrawl robot.

Use of the hybrid rigid-soft foot design showed significant improvement in robot functionality in terms of its compliance to all tested surfaces. This enhances the potential use of the robot in real-world onshore oil and gas pipeline inspection. Figure 14 shows a comparison between our robot and other state-of-the-art inchworm-inspired robots.

It is evident that our robot with its hybrid rigid-soft feet outperforms other robots in various functional parameters, particularly, its superior passive adaptability toward different diameter pipes. The other robots are either unable to do so or require active mechanisms, thereby needing more energy to adapt.

In the future, we will expand the hybrid rigid-soft foot development by incorporating an array of tactile sensors underneath the hybrid rigid-soft foot, enabling it to recognize the pipe diameter. This will make the robot more aware of toe deformation. The presented development brings the robot significantly closer to smooth crawling on various pipes in a real-world pipe-inspection scenario such as the concept shown here: http://www.manoonpong.com//EROFT/videoS2.mp4 (Khan et al., 2020).

## Data Availability

The original contributions presented in the study are included in the article/Supplementary Material, further inquiries can be directed to the corresponding authors.
