# Supplementary material for: Electromagnetic Feet With Soft Toes for Adaptive, Versatile, and Stable Locomotion of an Inchworm-Inspired Pipe Crawling Robot
Source: Front Bioeng Biotechnol. 2022 Feb 18;10:842816. doi: 10.3389/fbioe.2022.842816 (PMC8895823; doi:10.3389/fbioe.2022.842816)
Supplement: Supplementary file 1 [file DataSheet1.DOCX]

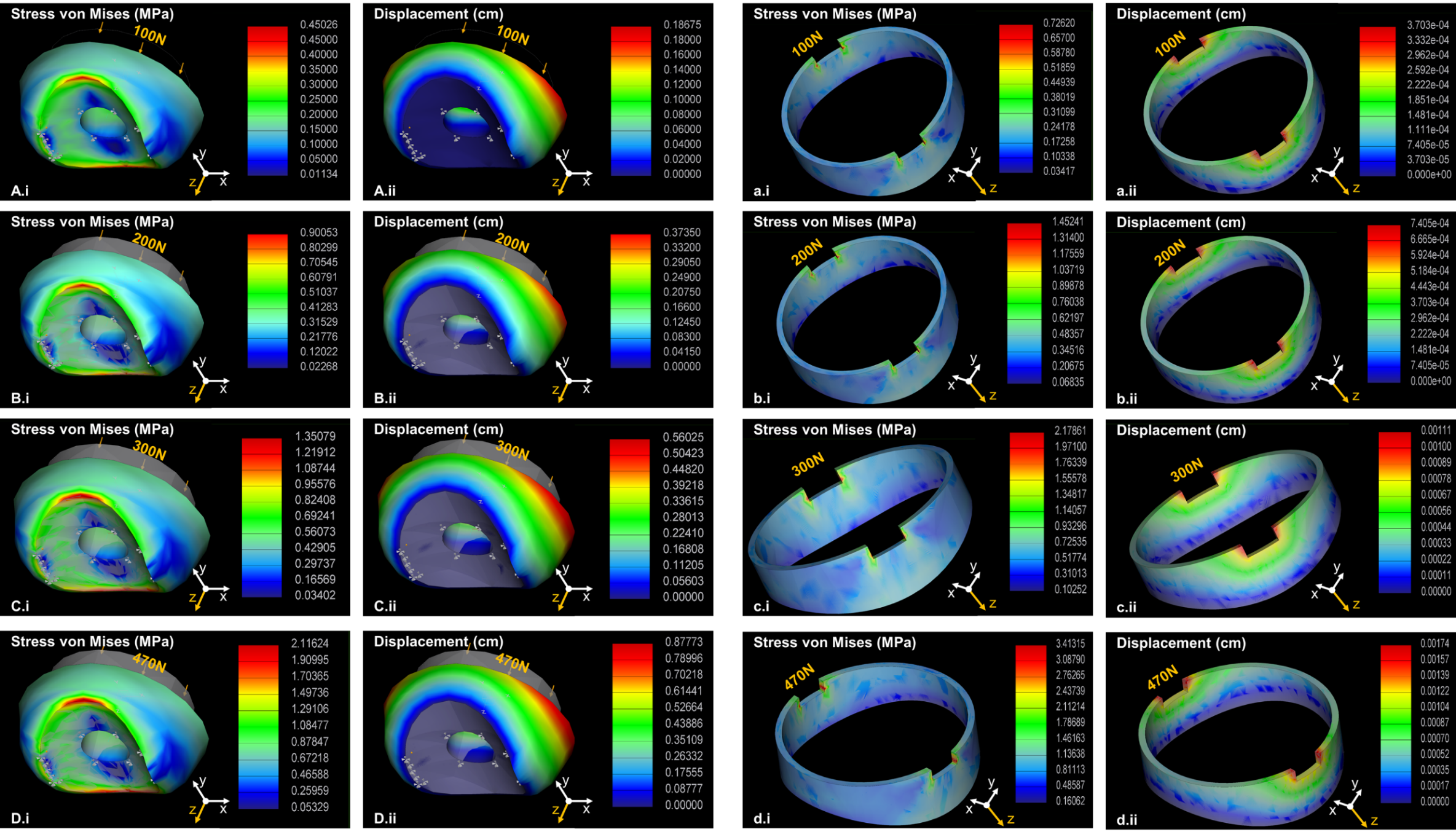
Supplementary Material

**Supplementary Figure 1.** An overview of FEA performed on the rigid toe using different loading conditions. a.i – d.i shows the von Mises stress under a set normal force/load acting from the top, whereas a.ii – d.ii shows the displacement under a set normal force/load acting from the top.


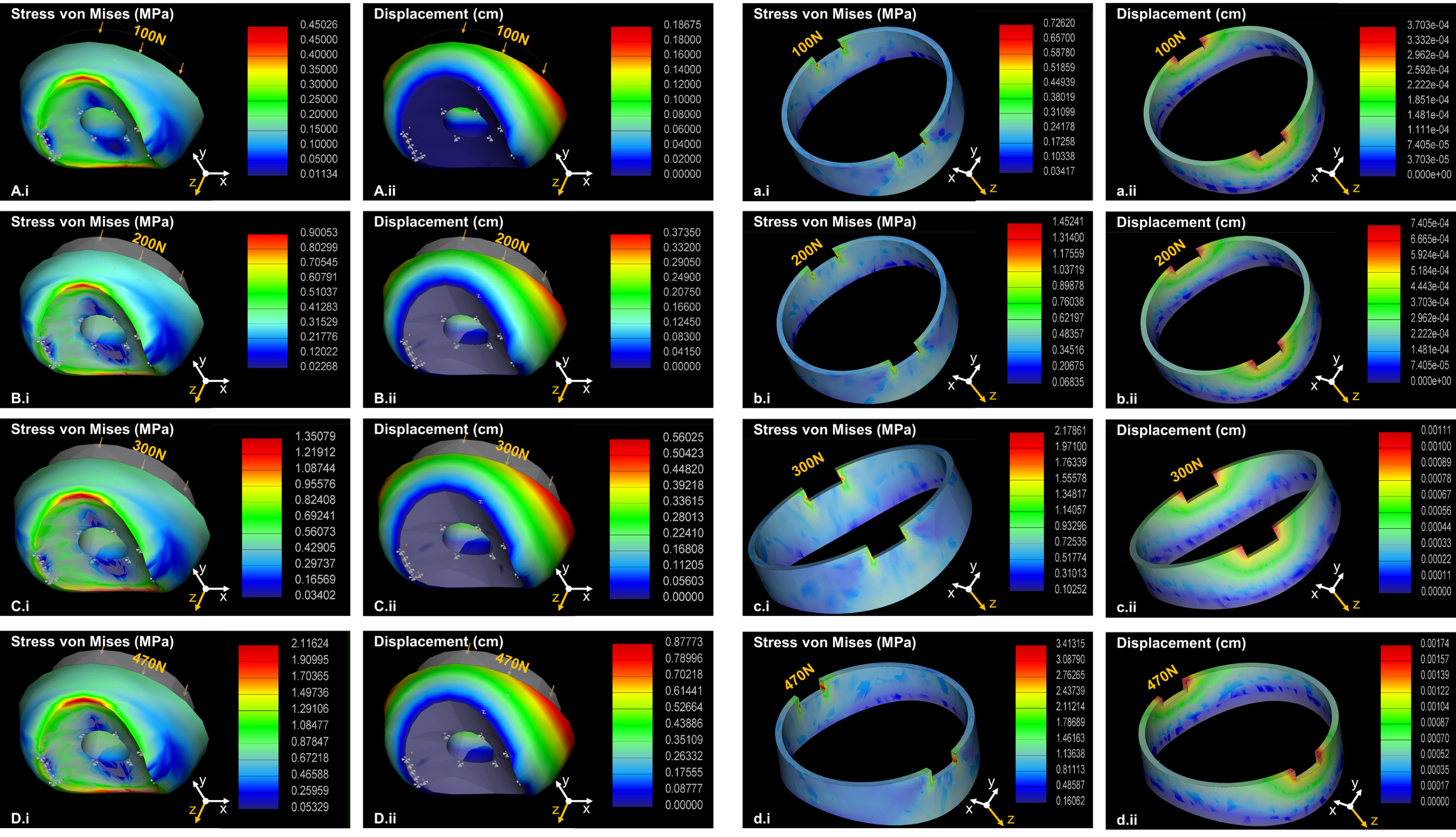
**Supplementary Figure 2.** An overview of FEA performed on the soft toe using different loading conditions. A.i – D.i shows the von Mises stress under a set normal force/load acting from the top, whereas A.ii – D.ii shows the displacement under a set normal force/load acting from the top.
